# Limited predictive value of achieving beneficial plasma (*Z*)-endoxifen threshold level by *CYP2D6* genotyping in tamoxifen-treated Polish women with breast cancer

**DOI:** 10.1186/s12885-015-1575-4

**Published:** 2015-08-01

**Authors:** Ewa E. Hennig, Magdalena Piatkowska, Jakub Karczmarski, Krzysztof Goryca, Elzbieta Brewczynska, Radoslaw Jazwiec, Anna Kluska, Robert Omiotek, Agnieszka Paziewska, Michal Dadlez, Jerzy Ostrowski

**Affiliations:** 1 Department of Gastroenterology, Hepatology and Clinical Oncology, Medical Center for Postgraduate Education, Warsaw, Poland; 2 Department of Genetics, Maria Sklodowska-Curie Memorial Cancer Center and Institute of Oncology, Warsaw, Poland; 3 Department of Breast Cancer and Reconstructive Surgery, Maria Sklodowska-Curie Memorial Cancer Center and Institute of Oncology, Warsaw, Poland; 4 Institute of Biochemistry and Biophysics, Polish Academy of Sciences, Warsaw, Poland; 5 Department of Internal Medicine and Oncology, Maria Sklodowska-Curie Memorial Cancer Center and Institute of Oncology, Warsaw, Poland; 6 Cancer Center-Institute, Roentgena 5, 02-781 Warsaw, Poland

**Keywords:** Tamoxifen, Breast cancer, CYP2D6, Polymorphism, Genotyping, Metabolite levels, Serum concentration, Mass spectrometry

## Abstract

**Background:**

Tamoxifen, the most frequently used drug for treating estrogen receptor-positive breast cancer, must be converted into active metabolites to exert its therapeutic efficacy, mainly through CYP2D6 enzymes. The objective of this study was to investigate the impact of *CYP2D6* polymorphisms on (*Z*)-endoxifen-directed tamoxifen metabolism and to assess the usefulness of *CYP2D6* genotyping for identifying patients who are likely to have insufficient (*Z*)-endoxifen concentrations to benefit from standard therapy.

**Methods:**

Blood samples from 279 Polish women with breast cancer receiving tamoxifen 20 mg daily were analyzed for *CYP2D6* genotype and drug metabolite concentration. Steady-state plasma levels of tamoxifen and its 14 metabolites were measured by using the ultra-performance liquid chromatography tandem mass spectrometry (UPLC-MS/MS) method.

**Results:**

In nearly 60 % of patients, including over 30 % of patients with fully functional CYP2D6, (*Z*)-endoxifen concentration was below the predefined threshold of therapeutic efficacy. The most frequently observed *CYP2D6* genotype was EM/PM (34.8 %), among which 83.5 % of patients had a combination of wild-type and *4 alleles. Plasma concentration of five metabolites was significantly correlated with *CYP2D6* genotype. For the first time, we identified an association between decreased (*E/*Z)-4-OH-*N*-desmethyl-tamoxifen-β-D-glucuronide levels (*r*^*2*^ = 0.23; *p* < 10^−16^) and increased CYP2D6 functional impairment. The strongest correlation was observed for (*Z*)-endoxifen, whose concentration was significantly lower in groups of patients carrying at least one *CYP2D6* null allele, compared with EM/EM patients. The *CYP2D6* genotype accounted for plasma level variability of (*Z*)-endoxifen by 27 % (*p* < 10^−16^) and for the variability of metabolic ratio indicating (*Z*)-endoxifen-directed metabolism of tamoxifen by 51 % (*p* < 10^−43^).

**Conclusions:**

The majority of breast cancer patients in Poland may not achieve a therapeutic level of (*Z*)-endoxifen upon receiving a standard dose of tamoxifen. This finding emphasizes the limited value of *CYP2D6* genotyping in routine clinical practice for identifying patients who might not benefit from the therapy. In its place, direct monitoring of plasma steady-state (*Z*)-endoxifen concentration should be performed to personalize and optimize the treatment.

**Electronic supplementary material:**

The online version of this article (doi:10.1186/s12885-015-1575-4) contains supplementary material, which is available to authorized users.

## Background

Tamoxifen is a selective estrogen receptor (ER) modulator commonly used for the treatment or prevention of hormone receptor-positive breast cancer, as well as for chemoprevention in women at high risk of developing breast cancer. Five years of tamoxifen treatment after surgery reduces the annual recurrence rate of ER-positive breast cancers by almost half and reduces the mortality rate by a third [1]. However, significant interindividual variability of tamoxifen efficacy is observed and disease recurs in 30–50 % of patients receiving adjuvant therapy [1]. Responsiveness to tamoxifen depends to a considerable degree on genetic variability in the level of patients’ drug-metabolizing enzymes.

Tamoxifen is a prodrug extensively metabolized via an enzymatic network predominantly including cytochrome P450 (CYP) isomers. At least 36 phase I tamoxifen metabolites have been recently described [2, 3]. The drug is primarily metabolized to *N*-desmethyl-tamoxifen (NDM-Tam), the most abundant metabolite in patients’ plasma, and 4-hydroxy-tamoxifen (4-OH-Tam), which are further converted to the secondary metabolite 4-hydroxy-*N*-desmethyl-tamoxifen (4-OH-NDM-Tam; endoxifen) [4]. Both endoxifen and 4-OH-Tam exhibit 30- to 100-fold higher anti-estrogenic potency than NDM-Tam or tamoxifen itself, with respect to their affinity for ER and suppression of estrogen-dependent breast cancer MCF7 cells proliferation, and are considered as the active tamoxifen metabolites responsible for the overall therapeutic drug activity [5–7]. Chemically, tamoxifen is a pure *Z*-isomer and its metabolites are formed primarily in the *Z*-form [8]. Recently, (*Z*)-isomers of endoxifen and 4-OH-Tam were found to exert the strongest ER inhibition among tamoxifen metabolites, with half maximal inhibitory concentration (IC50) values of 3 nmol/l and 7 nmol/l, respectively [2].

The major enzyme responsible for the conversion of tamoxifen to endoxifen and 4-OH-Tam is CYP2D6 [9]. There is great interindividual variability in CYP2D6 enzyme activity due to, at least in part, genetic polymorphisms in the *CYP2D6* gene. So far, over 140 allelic variants of *CYP2D6* have been described and a substantial part of these are associated with reduced or absent activity of the encoded enzyme [10]. With regard to the combination of carried alleles (*CYP2D6* genotype), each individual can be classified into one of four phenotypic groups: ultra-rapid- (UM), extensive- (EM) (wild type; wt), intermediate- (IM) or poor-metabolizer (PM) [11]. Patients with IM or PM phenotypes, represented by genetic variants associated with low or absent CYP2D6 activity, produced significantly less (*Z*)-endoxifen than wt allele carriers [9, 12, 13], and an increase in the plasma concentration of this metabolite was observed in accordance with the number of functional alleles [2]. Nonetheless, it is believed that *CYP2D6* genotypes explain no more than 40 % of the variability of steady-state concentration of (*Z*)-endoxifen [2]. Some concomitant medications, such as those used for the treatment of different mental disorders, have been shown to significantly inhibit CYP2D6, reducing overall production of active tamoxifen metabolites, especially in patients of the EM phenotype [13].

Several reports suggest that (*Z*)-endoxifen is the most potent tamoxifen metabolite in terms of its relative contribution to the drug’s anticancer effectiveness. Its anti-ER activity is comparable to that of (*Z*)-4-OH-Tam, but its plasma steady-state concentration in patients chronically treated with tamoxifen is several-fold higher than 4-OH-Tam [6, 9, 14]. Moreover, at the concentrations observed in CYP2D6 PM patients, the concentration-dependent endoxifen effects on ER degradation [15], global estrogen-induced gene expression in MCF7 cells [16], or tumor growth inhibition in a MCF7 xenograft bearing mouse model [17], were shown to have little or no effect.

Recently, a long-term tamoxifen treatment outcome study indicated that patients with (*Z*)-endoxifen plasma level below a threshold of 5.97 ng/ml may not benefit from the adjuvant therapy [12]. For patients with higher metabolite concentrations, a 26 % lower breast cancer recurrence rate was observed, defining for the first time a therapeutically beneficial threshold level of endoxifen. It is suggested that over 20 % of treated patients may not achieve sufficiently high endoxifen exposure [12, 18]. However, in CYP2D6 PM or IM patients, a daily dose increase from 20 mg to 30–40 mg of tamoxifen was found to significantly raise endoxifen concentrations to levels above or near the efficacy threshold [18–21]. As such, tamoxifen treatment outcomes might be improved by predicting which patients are likely to have low endoxifen levels and by ensuring the appropriate hormonal therapy regimen or dose accordingly.

The main objective of this study was to determine the *CYP2D6* allele and genotype frequencies and their associations with (*Z*)-endoxifen-directed tamoxifen metabolism in Polish breast cancer patients treated with the standard daily dose of 20 mg of tamoxifen. The steady-state plasma level of tamoxifen and its 14 metabolites were measured by a method of ultra-performance liquid chromatography tandem mass spectrometry (UPLC-MS/MS) and the metabolic ratio (MR) of (*Z*)-endoxifen concentration to the sum of the remaining measured compounds was correlated with the patients’ *CYP2D6* genotype. Unexpectedly, nearly 60 % of patients had (*Z*)-endoxifen plasma level below the 5.97 ng/ml efficacy threshold. In total, our findings emphasized the limited value of *CYP2D6* genotyping for the prediction of achieving therapeutic levels of (*Z*)-endoxifen in routine clinical practice. Rather, the direct measurement of steady-state (*Z*)-endoxifen plasma level should be performed to monitor the actual metabolite level for personalizing and optimizing the tamoxifen treatment.

## Methods

### Ethic statement

All enrolled patients were Polish Caucasians recruited at Maria Sklodowska-Curie Memorial Cancer Center-Institute of Oncology in Warsaw between mid-2012 and mid-2014. The local ethics committee approved the study and all participants provided written informed consent. The study protocol conforms to the ethical guidelines of the 1975 Declaration of Helsinki.

### Patients and blood sample collection

This study included 285 unselected women with verified hormone receptor-positive breast cancer who had taken tamoxifen at a standard 20 mg daily dose for at least one month to ensure steady-state blood concentration of drug and its metabolites. Selective serotonin reuptake inhibitors (SSRIs) intake was not a criterion for exclusion because information about SSRI uptake is not obligatorily recorded in routine clinical practice in Poland and therefore adequate data from medical databases was not available for objective monitoring. ER and progesterone receptor (PR) status were evaluated by enzyme immunoassay or immunohistochemistry (IHC). Human epidermal growth factor receptor 2 (HER2) expression was estimated by IHC and fluorescence in situ hybridization (FISH) methods. Nodal status was determined according to the International Union Against Cancer tumor-node-metastasis (TNM) classification.

Two peripheral blood samples taken from each patient were put into Monovettes® tubes containing K-EDTA as an anticoagulant (Sarstedt, Numbrecht, Germany), for plasma isolation, and for genomic DNA extraction and genotyping. Plasma was immediately separated by centrifugation and stored at −80 °C until UPLC-MS/MS analysis. Genomic DNA was extracted using QIAamp DNA Blood Mini Kit (Qiagen, Hilden, Germany), following the manufacturer’s protocol and quantified using a NanoDrop 1000 Spectrophotometer (Thermo Fisher Scientific, Waltham, MA, USA).

### *CYP2D6* genotyping

Isolated DNA samples were genotyped for *CYP2D6* alleles in accordance with the nomenclature available at http://www.cypalleles.ki.se/cyp2d6.htm [10]. To maximize CYP2D6 phenotype prediction, the individual TaqMan allelic discrimination assays were performed for 11 single-nucleotide polymorphisms (SNPs) and two TaqMan Gene Copy Number Assays for the gene deletion or duplication (Applied Biosystems, Foster City, CA, USA), according to manufacturer’s instructions. A SensiMix™ II Probe Kit (Bioline Ltd, London, United Kingdom) and a 7900HT Real-Time PCR system (Life Technologies, USA) were used for the assays. The measured allele and relevant SNP characteristics are presented in Additional file 1. Each allele was assigned to one of four phenotypic categories according to its associated enzyme function. PM (non-functional) alleles include: CYP2D6*3, *4, *5, *6, *7; IM (reduced function) alleles include: CYP2D6*9, *10, *17, *41; EM (wt; fully functional) alleles include CYP2D6*1 and *2; UM (increased function) alleles include: duplication of EM variants of the gene, such as CYP2D6*1XN and *2XN. Patients were assigned a *CYP2D6* genotype depending on the combination of alleles they carry, as PM/PM, IM/PM, IM/IM, EM/PM, EM/IM, EM/EM or EM/UM.

### Quantifying tamoxifen and its metabolites in plasma

Plasma concentration of tamoxifen and its 14 key metabolites: NDM-Tam, (*Z*)-endoxifen, (*E*)-endoxifen, 3-OH-NDM-Tam, 4′-OH-NDM-Tam, (*Z*)-4-OH-Tam, 3-OH-Tam, 4′-OH-Tam, (*Z*)-α-OH-Tam, (*E*)-α-OH-Tam, Tam-*N*-oxide, Tam-*N*-β-D-glucuronide (Tam-*N*-gluc), (*E/Z*)-4-OH-NDM-Tam-β-D-glucuronide ((*E/Z*)-4-OH-NDM-Tam-gluc), (*E*)-4-OH-Tam-O-β-D-glucuronide ((*E*)-4-OH-Tam-O-gluc) were measured using the UPLC-MS/MS method. All the above compounds, as well as deuterated internal standards (ISs): Tam-d5, NDM-Tam-d5 and (*Z*)-4-OH-Tam-d5, were purchased from Toronto Research Chemicals (North York, ON, Canada). For sample preparation and UPLC-MS/MS the following chemicals were used: acetonitrile, methanol and formic acid (99 %) from J. T. Baker (Avantor Center Valley, PA USA), ammonium formate from Fluka (Buchs, Switzerland), and ultrapure Milli-Q® water (Millipore Corp., Billerica, MA, USA).

Sample preparation was carried out as described by Dahmane et al. [22], with slight modifications, including protein precipitation by acetonitrile. Briefly, 100 μl of plasma was mixed with 300 μl of ISs solution (3.3 ng/ml of Tam-d5, 3.3 ng/ml of NDM-Tam-d5 and 4.2 ng/ml of (*Z*)-4-OH-Tam-d5) in acetonitrile. After vortexing and centrifugation (10 min; 18,000× *g*), 350 μl of clear supernatant was evaporated under nitrogen in 55 °C. Finally, samples were dissolved in 100 μl of 10 mM ammonium formate in 50 % methanol.

For sample separation, an Acquity UPLC system equipped with Acquity BEH Shield RP18 column (100 × 2.1 mm, 1.7 μm particle size) (Waters, Milford, MA, USA) was used. The column was thermostatted at 70 °C. The mobile phase A consisted of ultrapure water, and phase B consisted of 0.1 % formic acid in acetonitrile. The flow rate was 0.6 ml/min and the total run time was 16 min per sample under nonlinear gradient condition as follows: 10 % to 80 % B for 12 min (concave curve), 80 % B for 2 min, 95 % B for 1 min and 10 % B for 1 min.

Detection was performed on a triple quadrupole mass spectrometer Xevo TQ-MS (Waters) in the positive ion electrospray ionization mode, with general mass spectrometry (MS) parameters as follows: capillary voltage, 3 kV; desolvation temperature, 550 °C; desolvation gas (nitrogen) flow, 1000 l/h; cone gas (nitrogen) flow, 100 l/h and collision gas (argon) flow, 0.15 ml/min. Waters QuanLynx software was used for chromatograms integration and quantitation. The Additional file 2 details a description of the calibration method, the metabolite standards linearity range and the chosen MS/MS transition parameters.

### Statistical analyses

Mean concentration of tamoxifen and its metabolites was calculated for the seven functional *CYP2D6* genotype predicted categories: EM/UM, EM/EM, EM/IM, EM/PM, IM/IM, IM/PM and PM/PM. The Hardy-Weinberg equilibrium was checked using Chi-square test. Chi-square test was also used to qualitatively check the correlation of (*Z*)-endoxifen plasma level with the MR of (*Z*)-endoxifen to the sum of the remaining metabolites. The relevant categories were as follow: for (*Z*)-endoxifen - below/above 6 ng/ml; for MR - below/above 0.0146. The cutoff value of 0.0146 for the MR was estimated with simple linear regression fit for (*Z*)-endoxifen level and MR relationship.

Linear model (ordinary least squares) was fitted independently for ten metabolite concentrations and two MRs in six functional groups (indicator variables) with EM/EM as a reference to examine whether there was a linear association between the measured metabolite concentration and CYP2D6 genotype. Student’s *t* statistics was used to test if fitted coefficients were significantly different than 0. The Bonferroni correction was used to adjust *p*-value significance threshold for multiple comparisons and *p* < 6.9 × 10^−4^ (0.05/12 metabolites/6 genotypes) was considered significant. Calculations were performed using R statistical software package (http://www.r-project.org/) [23]. The *p*-value for Spearman rank correlation coefficient *r* was calculated by using the AS 89 algorithm and was used as a marker of significance of the association between metabolite plasma concentration and *CYP2D6* genotype. Squared *r* was used to indicate the proportion of variation of one variable which could be explained by the other variable.

## Results

### Patients

The plasma samples were obtained from 285 women with hormone receptor-positive breast cancer (median age at diagnosis 55, range 25-95) receiving standard treatment of 20 mg of tamoxifen daily. The median time period between the start of the tamoxifen treatment and taking a blood sample for analysis was 21.5 months (range 1–70). Among the enrolled patients, six had plasma drug concentrations of less than 10 % of the mean tamoxifen levels across all patients, and were excluded from the association analyses between *CYP2D6* genotype and tamoxifen metabolism. The clinical characteristics for the remaining 279 patients are listed in Table 1.

**Table 1:** Characteristics of patients

	Total *N = 279*
	Number of patients (%)
Age at diagnosis, median (range years)	55 (25 – 95)
Duration of tamoxifen treatment, median (range months)	21.5 (1 – 70)
Tamoxifen therapy	
neoadjuvant	7 (2.5)
adjuvant	258 (92.5)
metastasis	14 (5)
Breast cancer treatment	
surgery	276 (98.9)
received radiation	148 (53)
received chemotherapy	232 (83.2)
Hormonal status at diagnosis	
premenopausal	81 (29)
postmenopausal	115 (41.2)
unknown	83 (29.7)
Tumor size	
T1	105 (37.6)
T2	142 (50.9)
T3	20 (7.2)
T4	10 (3.6)
unknown	2 (0.7)
Node status	
N 0	132 (47.3)
N 1	112 (40.3)
N 2 - 3	30 (10.8)
unknown	5 (1.8)
Differential grade	
G1	39 (14)
G2	129 (46.2)
G3	67 (24)
unknown	44 (15.8)
Histology	
ductal	205 (73.5)
lobular	30 (10.8)
other	38 (13.6)
unknown	6 (2.2)
Hormone receptor status	
Estrogen+	269 (96.4)
Progesterone+	238 (85.3)
Both	230 (82.4)
HER2 status	
positive	64 (22.9)
negative	210 (75.3)
unknown	5 (1.8)

Tumor characteristics were at diagnosis. HER2: human epidermal growth factor receptor 2

### *CYP2D6* genotype frequency

The frequencies of measured *CYP2D6* alleles are indicated in Additional file 1. There was no deviation from the Hardy-Weinberg equilibrium for any genetic variant. Altogether, wt function alleles (*1, *2) were present in 60 % of enrolled patients. Among the alleles harboring impaired activity of the encoded enzyme, four with null activity (*4, *5, *6, *7) and four with reduced activity (*9, *10, *17, *41), the most frequently observed were alleles *4 (22.3 %) and *41 (7.7 %).

The appropriate *CYP2D6* genotype was assigned to 279 patients, who were further categorized into seven groups with respect to the encoded enzyme activity: EM/UM (6.5 %), EM/EM (31.2 %), EM/IM (15.4 %), EM/PM (34.8 %), IM/IM (1.1 %), IM/PM (3.9 %) and PM/PM (7.2 %) (Table 2). Among the most frequently occurring EM/PM genotype, 81 out of 97 patients (83.5 %) had a combination of alleles wt and *4.

**Table 2:** The *CYP2D6* genotype frequency

	Genotype	*N =* 279	Frequency (%)
EM/EM	*1/*2	44	15.8
*n =* 87 (31.2 %)	*1/*1	33	11.8
	*2/*2	10	3.6
EM/IM	*1/*41	24	8.6
*n =* 43 (15.4 %)	*2/*41	10	3.6
	*1/*10	7	2.5
	*2/*10	2	0.7
EM/PM	*1/*4	51	18.3
*n =* 97 (34.8 %)	*2/*4	30	10.8
	*1/*5	6	2.2
	*1/*3	4	1.4
	*2/*5	3	1.1
	*1/*6	1	0.4
	*2/*3	1	0.4
	*2/*6	1	0.4
IM/IM	*41/*41	1	0.4
*n =* 3 (1.1 %)	*10/*10	1	0.4
	*10/*41	1	0.4
IM/PM	*4/*41	6	2.2
*n =* 11 (3.9 %)	*4/*10	3	1.1
	*4/*17	1	0.4
	*5/*41	1	0.4
PM/PM	*4/*4	15	5.4
*n =* 20 (7.2 %)	*3/*4	2	0.7
	*4/*5	2	0.7
	*4/*6	1	0.4
UM	*1/*2 (xN)	10	3.6
*n =* 18 (6.5 %)	*1/*1 (xN)	5	1.8
	*2/*2 (xN)	3	1.1

*EM* extensive-metabolizer, *IM* intermediate-metabolizer, *PM* poor-metabolizer, *UM* ultra-rapid-metabolizer

### Tamoxifen and its metabolites plasma concentration

The plasma steady-state concentrations of tamoxifen and 14 of its metabolites were measured using UPLC-MS/MS. In the case of the two pairs of isobaric compounds, (*Z*)-endoxifen and 3-OH-NDM-Tam, and (*Z*)-4-OH-Tam and 3-OH-Tam, each of the respective metabolites were quantified as a sum because of inability to obtain distinct chromatographic separation between the two compounds. However, based on previous reports [2, 22, 24], the plasma concentrations of both 3-OH-Tam and 3-OH-NDM-Tam are several-fold lower than their 4-isomer counterparts and for simplicity, the respective measures were further referred to as purely (*Z*)-endoxifen and (*Z*)-4-OH-Tam concentrations. Remaining metabolites, including selected glucuronides, Tam-*N*-Oxide, 4′-OH-Tam and 4′-OH-NDM-Tam derivatives, as well as selected (*E*)- and (*Z*)-isomers, have been quantified separately (see Additional file 2 for the examples of sample calibration chromatograms).

For some compounds, we obtained a good signal-to-noise (S/N) ratio and clearly defined chromatography peaks at very low concentration. However, we were unable to obtain satisfactory linear calibration curves starting from such low levels. Thus, we decided to report the measures below the level of linearity range (LOLR), in cases where acceptable S/N ratio (>10) and the peak parameters were obtained, but these measures should be considered only as “estimated concentrations”. The linearity ranges of the calibration curve for standard metabolites are listed in Additional file 2.

The mean and median plasma concentrations with the range for each metabolite in 279 patients are included in Table 3. For tamoxifen and for most of its metabolites, a very wide variability of concentrations was observed between individuals. NDM-Tam and tamoxifen were the most abundant, with a range of 33.2–810.2 ng/ml (mean 235.5 ± 97.0) and 41.4–402.1 ng/ml (173.5 ± 67.7), respectively. Next, high levels of Tam-*N*-oxide (13.5 ± 7.4 ng/ml) were observed. As expected, of the two known clinically active metabolites, the mean concentration level of (*Z*)-endoxifen was higher than that of (*Z*)-4-OH-Tam (5.6 ± 3.3 *vs.* 2.5 ± 1.2 ng/ml). In agreement with other studies [2, 24], (*E*)-endoxifen was present only in trace amounts (below the LOLR) in the plasma of 30 patients (10.8 %). Similarly, the plasma concentration of (*Z*)-α-OH-Tam was within the calibration linearity range in only four samples, and was not detected at all in 116 patients (41.6 %). Trace amounts of (*E*)-α-OH-Tam were measured in the plasma of 271 patients, but in 239 of these (85.7 %) the estimated concentration was below the LOLR.

**Table 3:** The steady-state plasma concentration of tamoxifen and its metabolites

[ng/ml]	Mean	SD	Median	Min	Max	Not detected^a^
						*N = 279* (%)
Tamoxifen	173.53	67.68	169.76	41.4	402.10	0 (0)
NDM-Tam	235.53	97.04	220.53	33.20	810.16	0 (0)
(*Z*)-Endoxifen + 3-OH-NDM-Tam	5.55	3.26	4.90	0.55	18.23	0 (0)
(*Z*)-4-OH-Tam + 3-OH-Tam	2.46	1.20	2.45	0.12	5.66	0 (0)
(*E*)-Endoxifen	0.02	0.07	0	0.03	0.37	249 (89.2)
4′-OH-Tam	3.13	1.32	2.93	0.37	8.72	0 (0)
4′-OH-NDM-Tam	3.84	1.93	3.56	0.19	15.66	1 (0.4)
Tam-*N*-oxide	13.47	7.43	11.62	1.41	58.03	0 (0)
(*E*)-4-OH-Tam-O-gluc	0.23	0.17	0.18	0.03	1.19	1 (0.4)
(*E/Z*)-4-OH-NDM-Tam-gluc	1.12	1.02	0.88	0.10	11.49	0 (0)
(*E/Z*)-Tam-*N*-gluc	0.32	0.32	0.24	0.03	2.45	1 (0.4)
(*E*)-α-OH-Tam	0.36	0.17	0.37	0.02	1.02	8 (2.9)
(*Z*)-α-OH-Tam	0.04	0.03	0.04	0.02	0.19	116 (41.6)

^a^The number and (%) of patients with no detectable plasma concentration of compounds

### Association of *CYP2D6* genotype and tamoxifen metabolites concentration

**Fig. 1:**
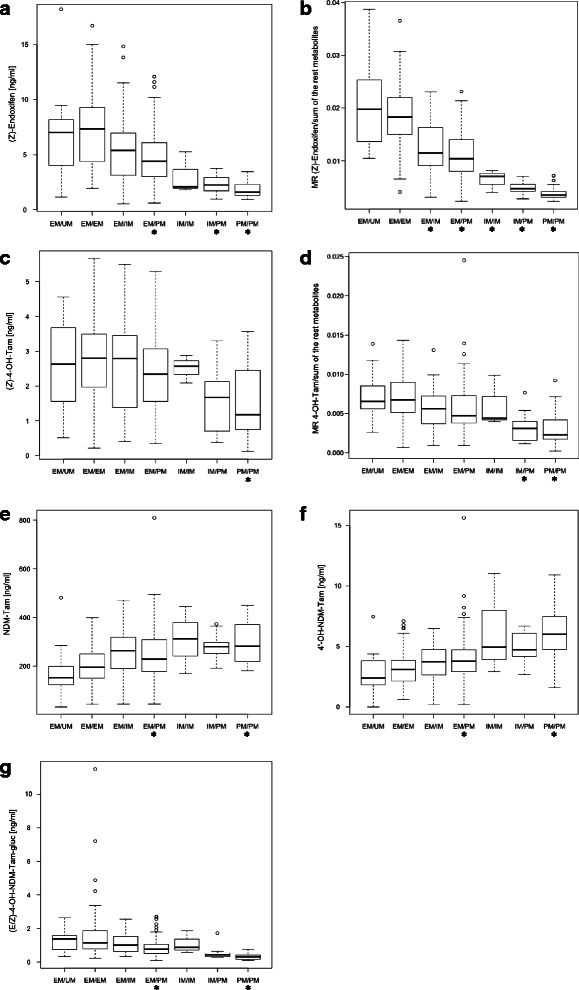
Significant association of the *CYP2D6* genotype with plasma concentration and molecular ratio of tamoxifen metabolites. **a** (*Z*)-endoxifen, **b** The MR of (*Z*)-endoxifen/sum of the remaining measured compounds, **c** (*Z*)-4-OH-tamoxifen, **d** MR of (*Z*)-4-OH-tamoxifen/sum of the remaining compounds, **e** *N*-desmethyl-tamoxifen, **f** 4′-OH-*N*-desmethyl-tamoxifen, **g** (*E/Z*)-4-OH-*N*-desmethyl-tamoxifen-β-D-glucuronide. The number of patients (N) was as follow: EM/UM (18), EM/EM (87), EM/IM (43), EM/PM (97), IM/IM (3), IM/PM (11), PM/PM (20). The horizontal line indicates the median plasma concentration, the box covers 25^th^-75^th^ percentiles and the maximum length of each whisker is 1.5× the interquartile range; dots outside the whiskers are outliers. Linear model (ordinary least squares) was fitted independently in six functional groups with EM/EM as a reference to examine whether there was an association between the measured metabolite concentration and CYP2D6 phenotype. Student’s *t* statistics was used to test if fitted coefficients were different than 0. The Bonferroni corrected *p-*value of less than 6.9 × 10^−4^ was considered significant. Significant associations are marked with asterisk (*)

Additional file 1 includes the list of measured plasma concentrations of analyzed compounds and assigned *CYP2D6* genotypes of all patients studied. The linear modeling indicated that the concentrations of five tamoxifen metabolites were significantly (*p* < 6.9 × 10^−4^) correlated with *CYP2D6* genotype (Fig. 1a, c, e-g). The level of NDM-Tam and 4′-OH-NDM-Tam tended to decrease in accordance with the number of functional enzyme alleles, showing significantly higher values for EM/PM and PM/PM genotypes, compared with EM/EM. Conversely, significantly lower concentrations of (*E/Z*)-4-OH-NDM-Tam-gluc were observed among EM/PM and PM/PM patients than in EM/EM patients.

The most evident association with *CYP2D6* genotype was observed for plasma (*Z*)-endoxifen concentration (Fig. 1a), the mean level of which was significantly decreased in PM/PM (1.8 ng/ml), IM/PM (2.3 ng/ml) and EM/PM (4.9 ng/ml) genotype carriers, as compared with EM/EM (7.3 ng/ml) carriers. The concentration of (*Z*)-4-OH-Tam, the other active metabolite, was significantly lower only in PM/PM patients (1.5 ng/ml *vs.* 2.7 ng/ml in EM/EM). The MR of (*Z*)-endoxifen plasma concentration to the sum of concentrations of the remaining measured compounds, which illustrates the direction of tamoxifen metabolism to (*Z*)-endoxifen production, showed a strong association with the *CYP2D6* genotype predicted functional group, increasing in accordance with the number of active alleles (Fig. 1b). For the MR of the levels of (*Z*)-4-OH-Tam and the sum of the remaining compounds, a much less pronounced association was indicated, although stronger than for steady-state (*Z*)-4-OH-Tam concentration (Fig. 1c, d).

The Spearman correlation coefficient indicated that 27 % (*p* < 10^−19^) of the variability in plasma levels of (*Z*)-endoxifen, and 51 % (*p* < 10^−43^) of the variability in its MR, was accounted for by the *CYP2D6* genotype. For (*Z*)-4-OH-Tam, the *CYP2D6* genotype accounted for only 5 % (*p* < 10^−3^) and 15 % (*p* < 10^−10^) variability in plasma level and corresponding MR, respectively. Additionally, 23 % (*p* < 10^−16^) of (*E/Z*)-4-OH-NDM-Tam-gluc, 18 % (*p* < 10^−12^) of 4′-OH-NDM-Tam and 13 % (*p* < 10^−9^) of NDM-Tam plasma level variability was associated with the *CYP2D6* genotype.

### Low (*Z*)-endoxifen concentration and the profile of tamoxifen metabolism

**Fig. 2:**
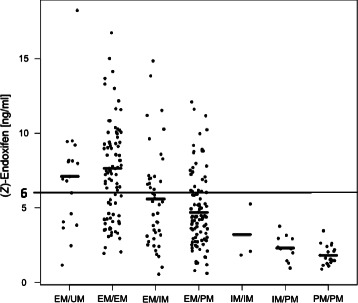
The steady-state concentration of plasma (*Z*)-endoxifen. The mean concentration of the metabolite in each *CYP2D6* genotype group and the predefine threshold level of 6 ng/ml are indicated

Based on UPLC-MS/MS analyzed plasma concentrations, the (*Z*)-endoxifen level was below the predefined threshold value of 5.97 ng/ml in 167 patients (59.9 %). There was a clear correlation (Chi-square, *p* = 3.8 × 10^−10^) between the number of patients with low active metabolite concentration and predicted CYP2D6 deficient functional category (Fig. 2). Among PM/PM, IM/PM and IM/IM genotype carriers, the measured (*Z*)-endoxifen plasma concentration was below the threshold level in all patients. Next, 61 % and 72 % of patients from EM/PM and EM/IM groups, and 33 % and 36 % of patients from EM/EM and EM/UM groups, respectively, exhibited (*Z*)-endoxifen levels below the threshold.

**Fig. 3:**
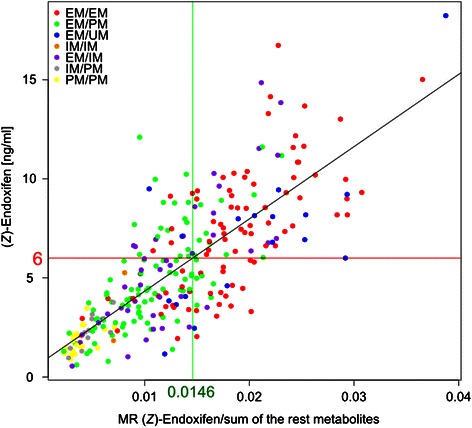
The comparison between plasma steady-state concentration of (*Z*)-endoxifen level and corresponding metabolic ratio. The metabolic ratio (MR) was estimated as (*Z*)-endoxifen plasma concentration divided by the sum of concentrations of the remaining measured compounds. The MR value of 0.0146 was delineated as the correlation coefficient with the level of 6 ng/ml. The assigned CYP2D6 functional category, based on *CYP2D6* genotype, is indicated in color

Correlation analysis between (*Z*)-endoxifen concentration and the corresponding MR revealed that the tamoxifen metabolism directed to the production of (*Z*)-endoxifen accounted for 61 % (*r =* 0.78, *p* = 10^−15^) variability of the absolute plasma level of (*Z*)-endoxifen. The MR value of 0.0146 was delineated as the threshold corresponding to the (*Z*)-endoxifen 6 ng/ml threshold level (Fig. 3). For 226 patients (81 %), the steady-state level of (*Z*)-endoxifen and MR values was consistent: for 84 women both values were above, and for 142 both values were below, the respective thresholds (6 ng/ml and 0.0146). In 25 patients (9 %) the level of (*Z*)-endoxifen was low (<6 ng/ml) despite the proper profile of tamoxifen metabolism (MR > 0.0146), and conversely, in 28 patients (10 %) the metabolite plasma level was > 6 ng/ml, although, the MR was below the threshold.

Altogether, our results of association analyses between *CYP2D6* genotype and (*Z*)-endoxifen concentration in the plasma, underlined the primary role of CYP2D6 in the profile of tamoxifen metabolism, specifically with respect to the production of NDM-Tam and (*Z*)-endoxifen. However, a significant proportion of active metabolite concentration depends on other genetic or environmental factors that alter the activity of the CYP2D6 enzyme or tamoxifen metabolism profile.

## Discussion

It is widely accepted that (*Z*)-endoxifen is the main active tamoxifen metabolite responsible for the overall clinical efficacy of the prodrug tamoxifen [12, 13, 19, 25, 26]. The plasma steady-state concentration of (*Z*)-endoxifen in drug-treated breast cancer patients is strongly associated with the number of functional *CYP2D6* alleles [2]. Recently, it has been suggested that the efficacy of tamoxifen therapy depends on meeting a threshold plasma level of (*Z*)-endoxifen rather than a linear dose-response effect [12]. Accordingly, the identification of patients who are unlikely to attain clinically sufficient plasma (*Z*)-endoxifen levels is of great interest for individual drug dose adjustment and therapy optimization.

To verify the usefulness of *CYP2D6* genotyping for the prediction of tamoxifen metabolism impairment, i.e., not achieving therapeutically beneficial (*Z*)-endoxifen exposure, we measured the steady-state concentration of tamoxifen and its metabolites in the plasma of nearly 280 Polish patients with breast cancer that were routinely treated with the standard 20 mg daily dose of the drug. Based on the patients’ *CYP2D6* alleles, all were classified into one of seven functional groups of the enzyme.

The *CYP2D6* allele and genotype frequencies among the studied patients were as expected for the Caucasian population, and similar to those obtained by others [2, 11, 19, 20, 27–29], with the most frequently occurring allele *4 (22.3 %), encoding the CYP2D6 enzyme of null activity (Additional file 1), and wt/*4 genotype (29.1 %) among the impaired activity enzyme carriers (Table 2). Compared with previous studies [2, 19, 27, 30], a slightly higher frequency of predicted EM/PM phenotype was observed among Polish patients (34.8 % *vs.* mean 23.8 %), but an average frequency of other functional groups, including PM/PM (7.2 % *vs.* 6.6 %), was consistent.

We adopted a sensitive UPLC-MS/MS method for the direct measurement of the drug and its metabolites in plasma [2, 22]. Originally, the method was developed for the quantitation of tamoxifen and seven of its metabolites, and, for the first time, 4′-OH derivatives were identified in the plasma of breast cancer patients [22]. In our study, we were successful in obtaining a perfect chromatographic separation of the drug and 12 of its metabolites, including both 4′-OH and α-OH derivatives, as well as Tam-*N*-oxide as an alternatives to the (*Z*)-endoxifen-directed pathway of tamoxifen metabolism. Moreover, three glucuronide conjugates were measured as phase II metabolite representatives. Consistent with previous reports, a wide range of concentrations of tamoxifen and of most of its primary metabolites were indicated (Table 3) [2, 12, 28].

**Fig. 4:**
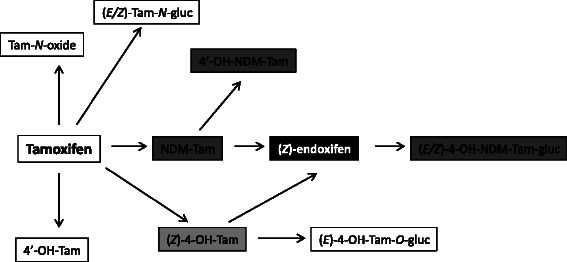
The (*Z*)-endoxifen-directed metabolism of tamoxifen according to the association of its metabolites concentration with *CYP2D6* genotype. The intensity of gray shading corresponds with the number of *CYP2D6* genotype predicted functional groups with a significant (*p* < 6.9 × 10^−4^) association with metabolite plasma concentration. White indicates no association and black indicates association with three genotypes, as indicated by linear modeling (see Fig. 1)

According to linear modeling analysis, the plasma concentrations of five metabolites were significantly (*p* < 6.9 × 10^−4^) correlated with the *CYP2D6* genotype (Fig. 1). For the first time, we have indicated an association between lower (*E/*Z)-4-OH-NDM-Tam-gluc levels (*r*^*2*^ = 0.23; *p* < 10^−16^) and an increasing degree of CYP2D6 functional impairment. In turn, consistent with other reports [12, 31], both NDM-Tam and 4′-OH-NDM-Tam were inversely correlated with the number of *CYP2D6* deficient alleles. Furthermore, both active metabolites tended to decrease in proportion to the degree of CYP2D6 deficiency, with the strongest correlation being observed for (*Z*)-endoxifen, whose concentration was significantly lower in groups of patients carrying at least one *CYP2D6* null allele, compared with EM/EM. In total, the *CYP2D6* genotype accounted for plasma level variability of (*Z*)-endoxifen and 4-OH-Tam by 27 % (*p* < 10^−16^) and 5 % (*p* < 10^−3^), respectively. However, the *CYP2D6* genotype accounted for 51 % (*p* < 10^−43^) of the variability of the MR for (*Z*)-endoxifen-directed drug metabolism. CYP2D6 deficiency has previously been shown to be associated with lower levels of both tamoxifen active metabolites [2, 12, 20, 27] and the correlation of plasma level variability with *CYP2D6* genotype was estimated to be 39 % for (*Z*)-endoxifen and 9 % for 4-OH-Tam [2]. Altogether, our results strongly confirmed the importance of CYP2D6 function in (*Z*)-endoxifen-directed metabolism of tamoxifen, as illustrated in Fig. 4 where the metabolic pathway responsible for the production of NDM-Tam and (*Z*)-endoxifen is highlighted by the strength of association of metabolite concentration with *CYP2D6* genotype.

From 36 known phase I metabolites of tamoxifen [3], only 3–4 are commonly estimated, including 4-OH-Tam and (*Z*)-endoxifen [12, 14, 27, 28, 32, 33]. Some recent studies have shown the importance of highly selective chromatographic separation for the accurate quantitation of measured compounds [22, 24, 34]. Several metabolites have molecular masses and fragmentation patterns similar to endoxifen and 4-OH-Tam, making them indistinguishable by MS. The lack of their proper liquid chromatography (LC) separation resulted in a factor of 2–3 times overestimation of active metabolite levels [24]. Although we managed to separate α-OH and 4′-OH derivatives of (*Z*)-endoxifen and 4-OH-Tam, we were unable to separate their 3-isomer counterparts. Nonetheless, the average concentration of (*Z*)-endoxifen in the plasma of Polish patients (5.6 ng/ml) was lower than in most other studies, where it ranged from 6.21 ng/ml to as high as 84.1 ng/ml [2, 13, 18, 28, 32, 33, 35, 36], some of which could possibly be falsely elevated because of peak coelution. The steady-state (*Z*)-endoxifen concentration might be related to the length of tamoxifen treatment. However, the median duration of tamoxifen therapy in our study was over 20 months, which seems to be more than long enough, considering the half-life of the drug in human plasma is 5–7 days [37].

The most unexpected result of our study was that in nearly 60 % of patients, the plasma concentration of (*Z*)-endoxifen was below the predefined 5.97 ng/ml level of clinical efficacy, including in over 30 % of patients with fully active CYP2D6 (EM/EM). Recently, it was suggested that slightly more than 20 % of women receiving a 20 mg dose of tamoxifen might not achieve high enough (*Z*)-endoxifen exposure [12, 18]. These divergent results may be due to the concomitant use of drugs that can affect CYP2D6 activity, especially among EM/EM patients. For example, some SSRIs, which are used in up to 30 % of breast cancer patients for depression or the relief of tamoxifen-induced menopausal symptoms such as hot flashes, were found to be strong CYP2D6 inhibitors [9, 13, 14]. Specifically, coadministration of paroxetine, an SSRI, was associated with a significant decrease in plasma endoxifen concentration, primarily in patients with the EM phenotype (by 58–72 %) [9, 13, 14]. It is now recommended that the use of strong CYP2D6 inhibitors be avoided where other treatment is possible.

The other critical factor for the overall steady-state (*Z*)-endoxifen level is the degree of compliance with tamoxifen therapy [26]. It has been estimated that only half of the patients remain on therapy after the 4th year [38]. Not adhering to the treatment was more commonly observed among patients with fully active CYP2D6 than those with null activity [39], most likely because they experience hot flashes more frequently [40]. This might help explain the observed low (*Z*)-endoxifen levels, despite having a MR over the threshold (Fig. 3), in some of EM/EM patients in our study.

Concomitant medications and compliance with the therapy were not reported in this study, which could be considered as a limitation. Our goal, however, was to estimate the predictive value of *CYP2D6* genotyping for achieving beneficial (*Z*)-endoxifen levels in routine clinical practice, where incomplete knowledge, or sparse data, on co-medication and self-reported adherence are frequent obstacles in the clinical setting.

Apart from CYP2D6, other drug-metabolizing enzymes may also contribute to the overall level of (*Z*)-endoxifen. For example, polymorphic *CYP2C9* was found to contribute to 4-OH-Tam production, which is the source for 20–30 % of total (*Z*)-endoxifen [2]. Also, the importance of *CYP3A5* [14] and *CYP2C19* [41] allelic variation has been suggested. In addition to the phase I metabolic enzymes, the pharmacokinetics of tamoxifen may also be influenced by phase II enzymes such as sulfotransferases (SULTs) or uridine 5′-diphospho-glucuronyltransferases (UGTs), which further metabolize active metabolites into hydrophilic derivatives that can be excreted. Genetic polymorphism of *SULT1A2* seems to play a role in maintaining optimal levels of both active tamoxifen metabolites, which are its substrates. The carriers of null *SULT1A2* enzyme alleles had significantly higher plasma concentrations of endoxifen and 4-OH-Tam [28], and specific combinations of *CYP2D6* and *SULT1A2* allelic variants seem to significantly affect an overall response to tamoxifen therapy [42]. Polymorphic variants of different UGTs were also described and were indicated to have the potential to alter elimination rates of endoxifen and 4-OH-Tam by prolonging or reducing their circulating half-lives [43]. The modifying impact of genetic polymorphism in other tamoxifen-metabolizing enzymes may account for the higher-than-expected (*Z*)-endoxifen level, based on MR value.

In summary, we have confirmed the importance of CYP2D6 activity in generating the overall (*Z*)-endoxifen plasma level, although additional factors also contribute. The mean steady-state plasma level of this active metabolite in breast cancer patients treated with tamoxifen was lower in our study than previously reported. In the majority of patients, the (*Z*)-endoxifen concentration was below the predefined level of therapeutic efficacy, suggesting that a significant number of patients in Poland may not benefit from the standard tamoxifen therapy.

## Conclusions

In making clinical decisions, it is important to identify patients who are likely to have insufficient (*Z*)-endoxifen concentration to benefit from the standard tamoxifen therapy. Admittedly, the *CYP2D6* genetic polymorphism has a major impact on (*Z*)-endoxifen level variation, but it might be significantly modified by several other unpredictable factors, such as concomitant use of CYP2D6 inhibitors or weak compliance. The lack of at least one fully functional *CYP2D6* allele may have a predictive value for not achieving a clinically effective level of (*Z*)-endoxifen among Polish patients, allowing us to consider increasing the drug-dosing regimen before the treatment starts. However, the true number of patients with low (*Z*)-endoxifen is much higher than predicted and includes some patients with fully active CYP2D6. In addition, increasing the dose of the drug does not necessarily mean the metabolite level will be increased, and this needs to be verified. Thus, the only reliable strategy to identify those individuals who may need dose adjustment or optimization of the treatment is the direct monitoring of (*Z*)-endoxifen concentration.

## Additional files

Additional file 1: **This file is in .xlsx format and includes:** **Table S1.** *CYP2D6* allele frequency. **Table S2.** CYP2D6 functional category and plasma concentration of tamoxifen and its metabolites in all studied patients. (XLSX 74 kb)
Additional file 2: **This file is in .docx format and includes additional data on the metabolites UPLC separation and mass spectrometry measurement.** (DOCX 125 kb)

## Abbreviations

CYP: Cytochrome P450
EM: Extensive-metabolizer
Endoxifen: 4-Hydroxy-*N*-desmethyl-tamoxifen
ER: Estrogen receptor
(*E*)-4-OH-Tam-O-gluc: (*E*)-4-Hydroxy-tamoxifen-O-β-D-glucuronide
(*E/Z*)-4-OH-NDM-Tam-gluc: (*E/Z*)-4-Hydroxy-*N*-desmethyl-tamoxifen-β-D-glucuronide
IM: Intermediate-metabolizer
IS: Internal standard
LC: Liquid chromatography
LOLR: Level of linearity range
MR: Metabolic ratio
MS: Mass spectrometry
NDM-Tam: *N*-Desmethyl-tamoxifen
4-OH-Tam: 4-Hydroxy-tamoxifen
PM: Poor-metabolizer
PR: Progesterone receptor
S/N: Signal-to-noise
SNP: Single-nucleotide polymorphism
SSRIs: Selective serotonin reuptake inhibitors
Tam-*N*-gluc: Tamoxifen-*N*-β-D-glucuronide
UM: Ultra-rapid-metabolizer
UPLC-MS/MS: Ultra-performance liquid chromatography tandem mass spectrometry
Wt: Wild type
