# Supplementary material for: Limited predictive value of achieving beneficial plasma (Z)-endoxifen threshold level by CYP2D6 genotyping in tamoxifen-treated Polish women with breast cancer
Source: BMC Cancer. 2015 Aug 1;15:570. doi: 10.1186/s12885-015-1575-4 (PMC4522133; doi:10.1186/s12885-015-1575-4)
Supplement: Additional file 2: — This file is in .docx format and includes additional data on the metabolites UPLC separation and mass spectrometry measurement. (DOCX 125 kb) [file 12885_2015_1575_MOESM2_ESM.docx]

## **Additional data file**

## **Metabolite standards calibration**

All compounds were divided between two calibration sets, A and B (Table 1), based on characteristic ions and retention times. Each set consisted of six calibration samples (CAL1 to CAL6) prepared by spiking blank plasma sample by an appropriate amount of analyte. The calibration range was chosen based on others’ published data, expected steady-state plasma metabolite concentration and our previous measurements. Linear calibration curves were obtained by plotting response (obtained by dividing peak area of analyte by peak area of internal standard) against calibration point of known concentration. Quality controls were performed by preparing spiked samples with analyte at different known concentrations (3 samples for each set – A and B).

Although for some compounds, we obtained a good signal-to-noise (S/N) ratio and clearly separated peaks at very low concentration, we were unable to obtain satisfactory linearity of calibration curves starting from such low levels. For example, for metabolites (*E*)-α-OH-Tam and (*Z*)-α-OH-Tam, an acceptable S/N ratio (>10) was obtained starting from 0.002 ng/ml, although the linearity range of calibration curves started from 0.6 and 0.1 ng/ml, respectively. Thus, we decided to limit the quantification of all compounds to our linearity range; however, we reported results that were below this level but with still acceptable peak parameters, as “estimated concentration.” The linearity range for each compound is listed in Table 1.

Table 1. Metabolite standards retention time, calibration set and linearity range.

| **Compound** | **Abbreviation** | **Retention time [min]** | **Calibration set** | **Linearity range [ng/ml]** |
| --- | --- | --- | --- | --- |
| Tamoxifen | Tam | 7.96 | A | 5 - 400 |
| N-desmethyl-tamoxifen | NDM-Tam | 7.87 | B | 10 - 1000 |
| (Z)-4-hydroxy-N-desmethyl-tamoxifen^*^ | Z-endoxifen | 6.94 | B | 0.6 - 100 |
| 3-hydroxy-N-desmethyl-tamoxifen^*^ | 3-OH-NDM-Tam | 6.96 | A | 0.6 - 100 |
| (Z)-4-hydroxy-tamoxifen^**^ | Z-4-OH-Tam | 7.04 | B | 0.1 - 100 |
| 3-hydroxy tamoxifen^**^ | 3-OH-Tam | 7.06 | A | 0.1 - 100 |
| (E)-4-hydroxy-N-desmethyl-tamoxifen | E-endoxifen | 6.81 | A | 0.6 - 100 |
| 4’-hydroxy-tamoxifen | 4’-OH-Tam | 7.28 | A | 0.6 - 100 |
| 4’-hydroxy-N-desmethyl-tamoxifen | 4’-OH-NDM-Tam | 7.19 | B | 0.6 - 100 |
| Tamoxifen-N-oxide | Tam-N-Ox | 8.19 | A | 0.6 - 100 |
| (E)-4-hydroxy-tamoxifen-O-β-D-glucuronide | 4-OH-Tam-O-Gluc | 6.17 | B | 0.1 - 100 |
| (E/Z)-4-hydroxy-N-desmethyl-tamoxifen-β-D-glucuronide^***^ | (E/Z)-4-OH-NDM-Tam-Gluc | 6.09  6.18 | A | 0.6 - 100 |
| Tamoxifen-N-β-D-glucuronide | Tam-N-Gluc | 8.56 | B | 0.1 - 100 |
| (Z)-α-hydroxy-tamoxifen | Z-a-OH-Tam | 5.97 | B | 0.1 - 100 |
| (E)-α-hydroxy-tamoxifen | E-a-OH-Tam | 5.32 | A | 0.6 - 100 |
| Tamoxifen-d5 | Tam-d5 | 7.96 | - | - |
| (Z)-4-hydroxy-tamoxifen-d5 | Z-4-OH-Tam-d5 | 7.04 | - | - |
| N-desmethyl-tamoxifen-d5 | NDM-Tam-d5 | 7.87 | - | - |

^*^, ^**^Two pairs of isobaric compounds for which we have not managed to obtain clear chromatographic separation. Respective two compounds from each pair were quantitated as a sum – (*Z*)-4-OH-Tam + 3-OH-Tam and (*Z*)-endoxifen + 3-OH-NDM-Tam.

^***^Although we were able to chromatographically separate E and Z isoforms of 4-OH-NDM-Tam-Gluc, we quantitated these two compounds together because we could obtain analytical standard only of a mixture of both isomers.

**MS/MS transition parameters**

Each compound was tested separately to choose optimal fragmentation and sensitivity. The chosen parent ions, fragmentation ions and collision energies are summarized in Table 2.

Because of their structural similarity, some compounds have the same characteristics of their ion pairs, which make them indistinguishable for MS. This means that proper LC separation is the key point in obtaining reliable results. When only some tamoxifen metabolites are measured, retention times of other isobaric compounds need to be checked to avoid falsely elevated results caused by peak coelution.

Table 2. MS/MS transitions parameters.

| **Compound** | **Parent ion (m/z)** | **Fragment ion (m/z)** | **Collision energy (eV)** |
| --- | --- | --- | --- |
| Tam | 372.25 | 72.10 | 22 |
|  |  | 129.13 | 22 |
| Tam-N-Ox | 388.23 | 72.25 | 20 |
|  |  | 129.05 | 30 |
| Tam-N-Gluc | 548.50 | 72.25 | 30 |
|  |  | 372.10 | 18 |
| 4-OH-Tam-O-Gluc | 564.26 | 72.25 | 30 |
|  |  | 388.20 | 26 |
| 4’-OH-Tam | 388.23 | 72.25 | 22 |
|  |  | 129.05 | 30 |
| Z-4-OH-Tam | 388.23 | 72.25 | 30 |
|  |  | 129.05 | 30 |
| 3-OH-Tam | 388.23 | 72.25 | 30 |
|  |  | 129.05 | 30 |
| Z-a-OH-Tam | 388.23 | 72.25 | 18 |
|  |  | 129.05 | 16 |
| E-a-OH-Tam | 388.23 | 72.25 | 18 |
|  |  | 129.05 | 16 |
| Z-endoxifen | 374.21 | 58.05 | 20 |
|  |  | 129.10 | 20 |
| 3-OH-NDM-Tam | 374.21 | 58.05 | 26 |
|  |  | 129.10 | 22 |
| E-endoxifen | 374.21 | 58.05 | 20 |
|  |  | 129.10 | 20 |
| NDM-Tam | 358.22 | 58.11 | 25 |
|  |  | 129.10 | 20 |
| (E/Z)-4-OH-NDM-Tam-Gluc | 550.24 | 374.24 | 20 |
| 4’-OH-NDM-Tam | 374.21 | 58.05 | 30 |
|  |  | 129.10 | 30 |
| Tam-d5 | 377.25 | 72.10 | 22 |
|  |  | 134.08 | 22 |
| Z-4-OH-Tam-d5 | 393.23 | 72.25 | 30 |
|  |  | 236.33 | 30 |
| NDM-Tam-d5 | 363.22 | 58.11 | 25 |
|  |  | 134.23 | 25 |

**Representative chromatograms of calibration sample**


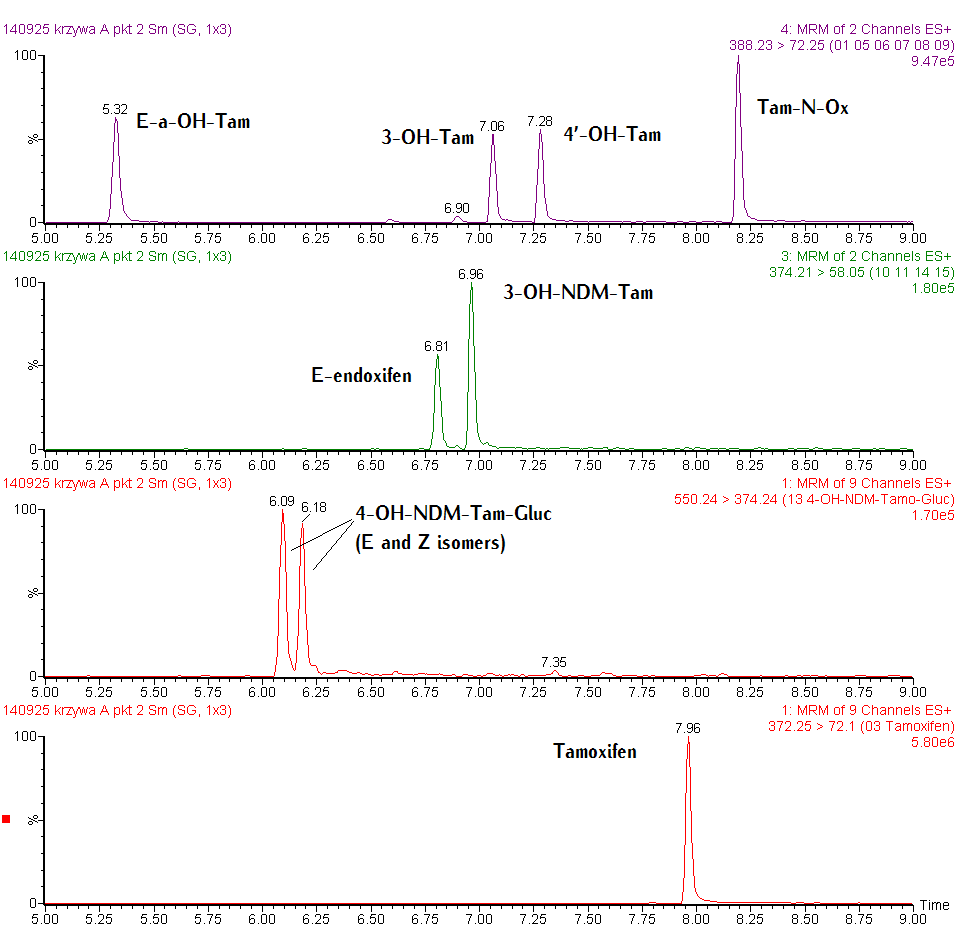


Figure 1. Representative chromatogram of second calibration point (CAL2A) from calibration set A (30 ng/ml for Tamoxifen and 3 ng/ml for other compounds).


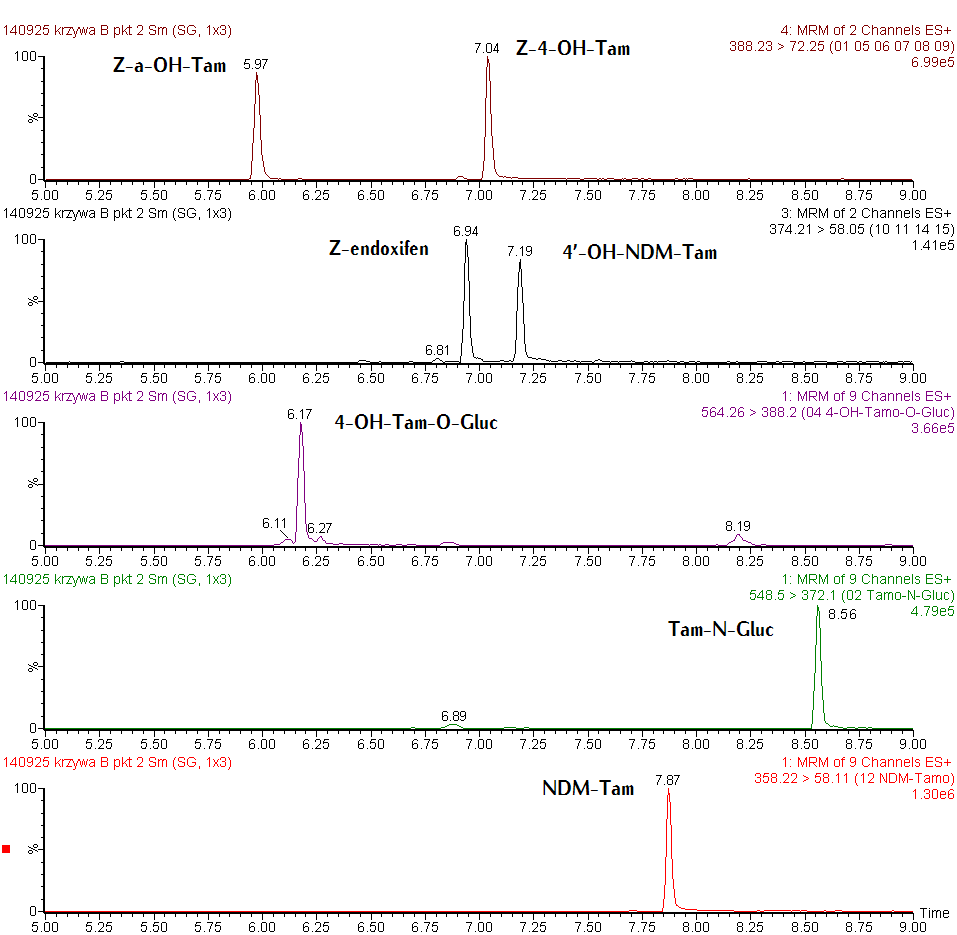


Figure 2. Representative chromatogram of second calibration point (CAL2B) from calibration set B (50 ng/ml for NDM-Tam and 3 ng/ml for other compounds).


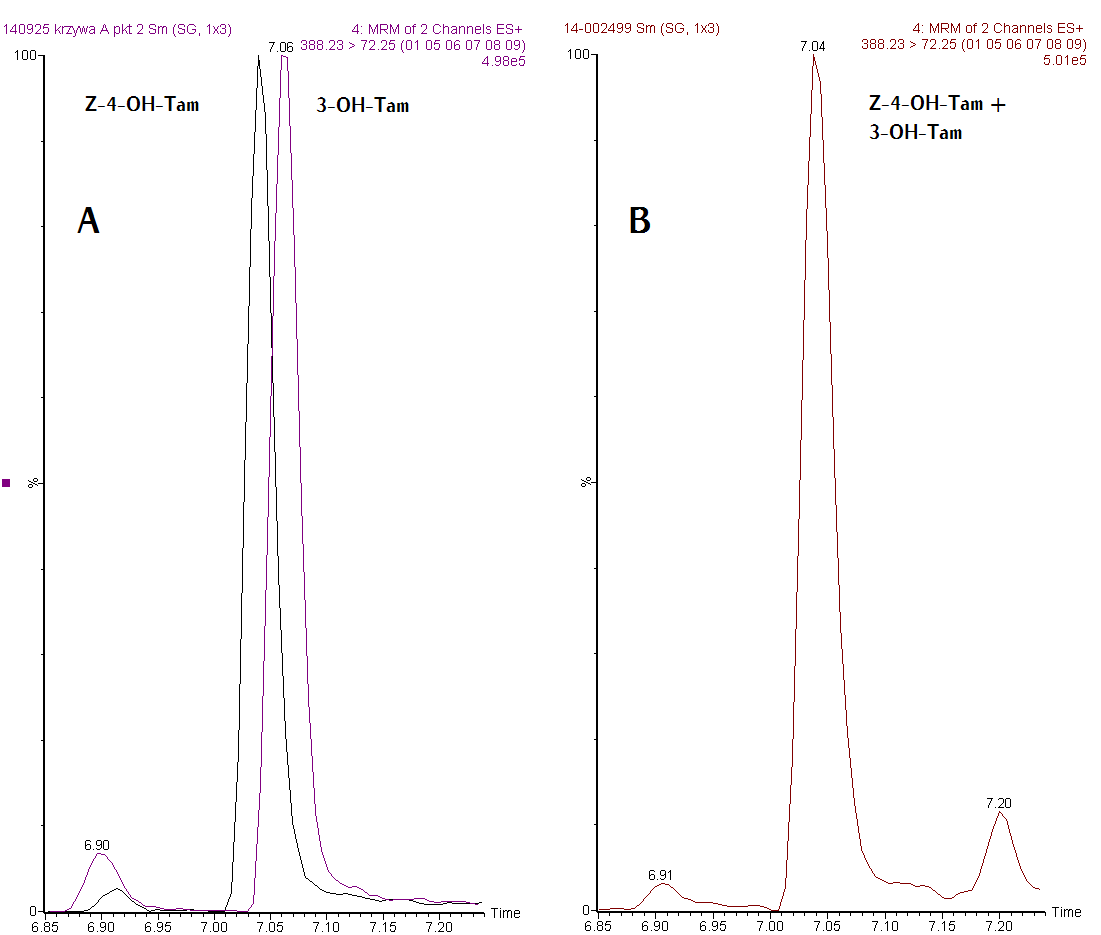


Figure 3. A) Overlapped chromatogram of CAL2A and CAL2B. B) An actual patient sample chromatogram showing peak of (*Z*)-4-OH-Tam together with 3-OH-Tam.


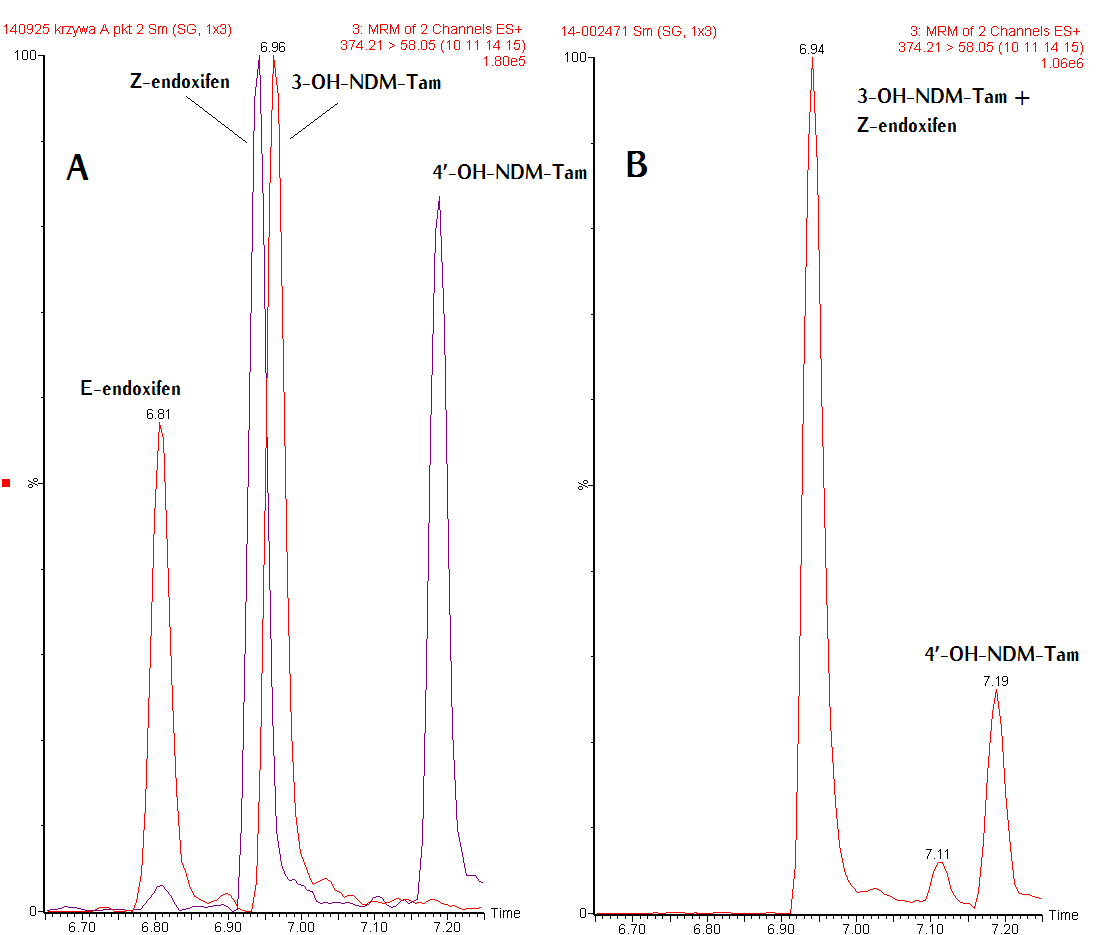


Figure 4. A) Overlapped chromatogram of CAL2A and CAL2B. B) An actual patient sample chromatogram showing peak of (*Z*)-endoxifen together with 3-OH-NDM-Tam.
